# Discussing diet, nutrition, and body weight after treatment for gynecological cancer: a conversation analytic study of outpatient consultations

**DOI:** 10.1007/s11764-023-01345-w

**Published:** 2023-03-10

**Authors:** Elizabeth A. Johnston, Stuart Ekberg, Bronwyn Jennings, Nisha Jagasia, Jolieke C. van der Pols

**Affiliations:** 1 Cancer Council Queensland, Fortitude Valley, Brisbane, QLD Australia; 2 Faculty of Health, School of Exercise and Nutrition Sciences, Queensland University of Technology (QUT), Kelvin Grove, Brisbane, QLD Australia; 3 QIMR Berghofer Medical Research Institute, Population Health Program, Herston, QLD Australia; 4 Faculty of Health, School of Psychology and Counselling, Queensland University of Technology (QUT), Kelvin Grove, Brisbane, QLD Australia; 5 Department of Gynaecological Oncology, Mater Hospital Brisbane, South Brisbane, QLD Australia

**Keywords:** Cancer survivors, Conversation analysis, Doctor-patient interaction, Endometrial cancer, Malnutrition, Ovarian cancer

## Abstract

**Purpose:**

To generate direct observational evidence for understanding how diet, nutrition, and weight-related topics are discussed during follow-up after treatment for gynecological cancer, as recommended by survivorship care guidelines.

**Methods:**

Conversation analysis of 30 audio-recorded outpatient consultations, involving 4 gyne-oncologists, 30 women who had completed treatment for ovarian or endometrial cancer, and 11 family members/friends.

**Results:**

From 21 instances in 18 consultations, diet, nutrition, or weight-related talk continued beyond initiation if the issue raised was ostensibly relevant to the clinical activity being undertaken at the time. These instances led to care-related outcomes (i.e., general dietary recommendations, referral to support, behavior change counseling) only when the patient identified needing further support. Diet, nutrition, or weight-related talk was not continued by the clinician if it was not apparently related to the current clinical activity.

**Conclusions:**

The continuation of diet, nutrition, or weight-related talk during outpatient consultations after treatment for gynecological cancer, and the subsequent delivery of care-related outcomes, depends on its immediate clinical relevance and the patient indicating needing further support. The contingent nature of these discussions means there can be missed opportunities for the provision of dietary information and support post-treatment.

**Implications for Cancer Survivors:**

If seeking information or support for diet, nutrition, or weight-related issues post-treatment, cancer survivors may need to be explicit regarding their need for this during outpatient follow-up. Additional avenues for dietary needs assessment and referral should be considered to optimize the consistent delivery of diet, nutrition, and weight-related information and support after treatment for gynecological cancer.

**Supplementary Information:**

The online version contains supplementary material available at 10.1007/s11764-023-01345-w.

## Introduction

Improving communication between patients and healthcare providers has been identified as a research priority for cancer survivorship care in many countries including Australia and the USA [1, 2]. As cancer survivorship increases with earlier detection and advances in cancer treatment [3], so does the need for high-quality care post-treatment to maximize wellness and reduce the risk of cancer recurrence and comorbid disease. For this reason, national cancer organizations endorse information and support for healthy lifestyle behaviors and symptom management as critical components of survivorship care [4, 5]. This study considers the provision of diet, nutrition, and weight-related information and support to cancer survivors, through a focus on gynecological cancer survivors.

Gynecological cancer includes cancers of the female reproductive tract, the most prevalent in Australia being endometrial and ovarian [6]. Among these cancer types, comorbid disease, including obesity and diabetes, has been associated with poorer survival [7, 8]. Dietary intervention post-treatment has been associated with improved diet quality and weight status among overweight and obese endometrial cancer survivors [9]. For women with ovarian cancer, one in three report ongoing physical symptoms after primary treatment, including fatigue, poor appetite, early satiety, and bowel disturbances [10]. These symptoms may require dietary support if food intake becomes consistently inadequate to meet requirements [11, 12]. Thus, diet and weight-related information and support post-treatment could reduce morbidity and mortality in this population [13].

In previous studies, gynecological oncology clinicians report their willingness to facilitate healthy lifestyle discussions and access to supportive care for women who have completed treatment for gynecological cancer [14, 15]. However, in practice, clinicians report several barriers to these discussions, including limited consultation time, insufficient training, lack of clear referral pathways, and uncertainty regarding the efficacy of counseling [14, 15].

Notwithstanding the apparent challenges clinicians report in promoting discussions about diet, nutrition, and weight, studies among gynecological cancer survivors suggest that these discussions may be well received during post-treatment follow-up [16–19]. This includes a preference for direct communication with healthcare professionals about these topics and referral to support services, as recommended by survivorship care guidelines and optimal care pathways [4, 5]. However, previous studies suggest that diet and weight-related discussions do not routinely occur during post-treatment consultations [20], referral to dietary support services is limited [10, 18], and gynecological cancer survivors commonly report seeking diet and weight-related information from online or media sources [21, 22].

Overall, findings from existing research suggest there may be a disconnect between recommendations for diet, nutrition, and weight-related communication after treatment for gynecological cancer and survivorship care in practice. To bridge this gap, there is a need to identify what actually happens in clinical practice. To date, studies investigating health behavior talk in gynecological cancer survivorship settings have utilized interviews or surveys [14–19]. However, self-report methods are limited by participants’ recall of events and do not capture the precise ways in which clinicians and patients communicate [23, 24]. Additionally, previous studies of health behavior communication in medical settings have identified that contextual features, such as prior and subsequent talk in conversation, are relevant to the investigation of effective communication practices [25–28].

To avoid limitations of self-report data, this study aims to generate direct observational evidence for (1) understanding how diet, nutrition, and weight-related topics are discussed during follow-up after treatment for gynecological cancer, and (2) exploring whether there are challenges associated with enacting survivorship care guidelines for these discussions in clinical practice. These aims are important for supporting gynecological oncology clinicians and cancer survivors to engage in best-practice survivorship care.

## Methods

### Setting and participants

Data collection was conducted in the gynecological oncology outpatient department of a large public hospital in Australia over a 7-month period. Gynecological oncologists (hereafter referred to as “gyne-oncologists”) were eligible to participate if they had completed, or were in the process of completing, sub-specialty training. Patients were eligible to participate if they had completed treatment within the past 12 months for a confirmed endometrial or ovarian malignancy and were attending an outpatient appointment with a participating gyne-oncologist, aged 18 years or older, English-speaking, able to provide informed consent, and not receiving end-of-life care. Time since treatment completion was limited to a maximum of 12 months because this study aimed to investigate dietary communication in the early post-treatment phase.

This study was approved by the Human Research Ethics Committee (HREC) of the hospital, with administrative approval subsequently provided by the HREC at Queensland University of Technology (Approval #2000000829). Written informed consent was obtained from all participants including gyne-oncologists, patients, and accompanying family members or friends. Participants were informed that consultations would be recorded to study communication about supportive care. All participants authorized publication of transcripts of the audio recordings collected for this study. To protect participants’ privacy and confidentiality, all names, places, and other potentially identifying references have been anonymized.

### Data collection

All eligible women with an appointment at the outpatient clinic during the data collection period were invited to participate in the study. Of 57 eligible women, 46 attended their appointment and 34 (74%) consented to participate (see Online Resource 1). Of those who consented, 30 consultations were able to be recorded, creating a data corpus involving four gyne-oncologists (one consultant, three fellows), 30 patients (19 endometrial cancer survivors, 11 ovarian cancer survivors), and 11 accompanying persons. On average, patients were aged 57 years (range 21 to 83 years) and were 6 months post-treatment (range 2 to 11 months). Further details on patient characteristics by cancer type are provided in Online Resources 2 and 3.

Consultations were audio-recorded by the gyne-oncologist. Video recording of the consultations would have enabled analysis of the multimodal aspects of communication, such as posture, gaze, and facial expressions [29]. The logistics of collecting recordings in a busy clinical environment and the need to minimize participant burden necessitated audio recording only. On average, consultations were 19 minutes in length (range 8 to 39 min). None of the researchers were present during the consultation. Key disease and treatment-related information for each participant were extracted from patient medical records by a member of the research team employed at the hospital and documented on a standardized form developed for this study.

Following the consultation, the first author contacted patients by telephone to complete a 16-item questionnaire to collect information on sociodemographic characteristics, current health behaviors, physical well-being, and diet and weight-related support post-treatment. Questions related to physical well-being were extracted from the Patient-Generated Subjective Global Assessment (PG-SGA) [30]. As this questionnaire could prime participants about the specific focus of the study, patients with a subsequent consultation during the data collection period were not invited to participate again in the study. Gyne-oncologists were not informed of the specific focus of the study until after data collection was completed to avoid influencing communication practices. In accordance with ethical guidelines [31], this limited disclosure was approved by the HREC.

### Analytic approach

To overcome the limitations of self-report methods, this study utilized conversation analysis, a leading approach for studying real-world communication [32], with four decades of application in healthcare settings [23, 24, 33]. Its methodology is grounded in direct observation and involves collecting recordings of naturally occurring conversations and developing specialized transcriptions of these recordings to understand how people perform social actions, such as the delivery and receipt of healthcare, through talk [32].

The first author reviewed the audio-recorded consultations to identify when diet, nutrition, or body weight-related topics were mentioned. Where diet, nutrition, or weight-related talk occurred intermittently throughout the consultation, these were considered one instance of talk if they were topically connected by participants. Where diet, nutrition, or weight-related talk on different topics occurred throughout the consultation, these instances were considered separate instances of talk since they did not have topical connection. For example, talk about how to manage diarrhea using dietary strategies and talk about weight loss were considered separate instances of diet, nutrition, and weight-related talk. The instances identified were then transcribed verbatim, including several turns of prior and subsequent talk.

To facilitate detailed analysis, verbatim transcripts were further transcribed using the Jefferson Transcription System, to capture the verbal and non-verbal details of conversation (see Appendix Table 1 for transcription conventions) [34]. For example, square brackets are used to mark overlap of talk between two speakers and a comma or period are used to indicate a speaker’s shift in intonation (up or down). A key premise of conversation analysis is that utterances in conversation are influenced by the mutual monitoring of the others’ talk; therefore, these details, such as overlap and subtle shifts in intonation, can have interactional consequences for how the conversation unfolds [34]. The detailed transcripts were then analyzed on a case-by-case basis, in consultation with the second author. This collaborative approach is a key part of conversation analytic methodology and central to its rigor [35]. The analysis sought to identify and describe how diet, nutrition, or weight-related conversations were initiated by gyne-oncologists and patients, and the interactional consequences of these practices (i.e., how diet, nutrition, or weight-related talk is constructed by both the clinician and patient following its initiation). The analysis is presented below using transcribed fragments to illustrate analytic findings.

## Results

**Fig. 1:**
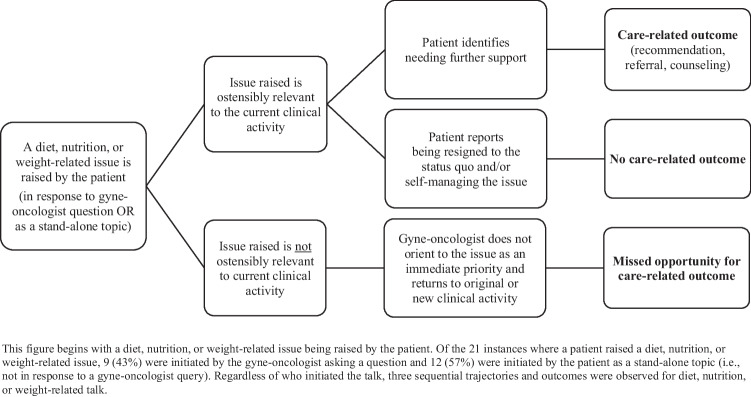
Three sequential trajectories and outcomes of diet, nutrition, or weight-related talk observed during outpatient follow-up after treatment for gynecological cancer

A diet, nutrition, or weight-related conversation occurred in 18 of the 30 consultations recorded. Across these 18 consultations, there were 21 instances of diet, nutrition, or weight-related talk; these topics were mostly raised once and never more than twice, in a consultation. In all 21 instances, the patient identified a diet, nutrition, or weight-related issue; 9 (43%) of the issues raised were in response to a gyne-oncologist query and 12 (57%) were initiated by the patient as a stand-alone topic (i.e., not in response to a gyne-oncologist query). Analysis of the 21 instances of diet, nutrition, or weight-related talk identified three sequential trajectories and outcomes of this talk (Fig. 1). These three trajectories and outcomes are presented below and appeared to occur irrespective of who initiated the discussion or the patient’s cancer type. Finally, a family member or friend was present in eight of the 18 consultations with diet, nutrition, or weight-related talk but were rarely involved in these conversations.

### Diet, nutrition, or weight-related talk sustained and care-related outcomes accomplished

Fragments 1 to 3 illustrate instances where diet, nutrition, or weight-related talk continued beyond initiation to accomplish care-related outcomes. The following sequence of actions was observed in these and other instances that followed the same trajectory (beginning at 1.2 if patient initiated):

1.1 Gyne-oncologist inquires about potential treatment late effects, signs of cancer recurrence, or additional concerns.
  1.2 Patient reports a diet, nutrition, or weight-related issue post-treatment, that is (a) directly relevant to the clinical activity initiated by the gyne-oncologist’s inquiry, or (b) introduced by the patient as a stand-alone topic. The patient then identifies needing further support in one of three ways:
i. Asking a question about normality or ongoing management,
ii. Orientating to the possibility of referral to support, or
iii. Reporting that current strategies are not working and uncertainty as to why this is the case.
1.3 Gyne-oncologist provides general dietary recommendations if in response to (i), referral to support if in response to (ii), or behavior change counseling if in response to (iii).

The first fragment is an instance where diet-related talk is sustained and leads to general dietary recommendations as a care outcome. It begins with a question from the gyne-oncologist to solicit additional concerns from the patient, a common practice used to transition from the “business” of a medical consultation towards the possibility of closing the consultation [36].

Fragment 1 [G01, P01, 24:21–26:57]

**Table:**
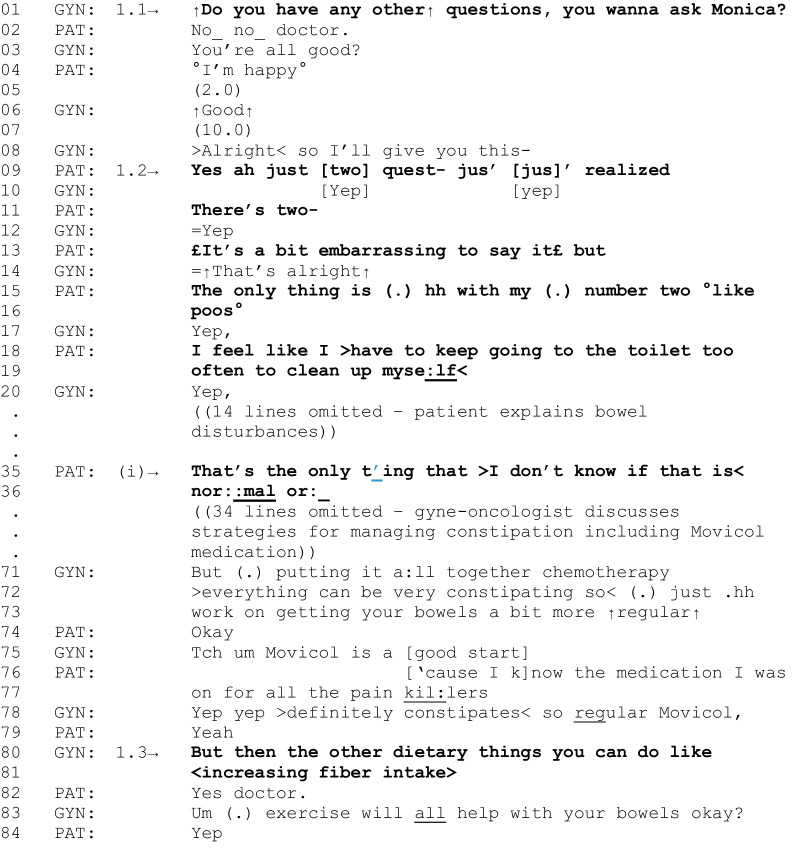


*Patient characteristics: ovarian cancer, obese, intending to lose weight*

In Fragment 1, the gyne-oncologist asks the patient if they have “any other questions” (line 1). The gyne-oncologist’s use of “any” suggests that the preferred response (i.e., expected answer) to the query is “no” [37]. This is because “any” is a negative polarity item, meaning its use only makes sense in a negative grammatical context (in this case, “No, I don’t have any other questions”) rather than a positive grammatical context (e.g., “Yes, I do have any other questions”) [37]. This preferred response is subsequently delivered by the patient in line 2 (“No, no doctor”) and confirmed again by the gyne-oncologist and patient in lines 3–6. However, as the gyne-oncologist moves to conclude the consultation with “alright,” which closes down this activity [38, 39], the patient identifies that they do in fact have two questions (lines 8–14). This sequence mirrors a phenomenon observed in primary care consultations, known as the “doorknob concern,” where patients defer the initiation of a priority concern until a point where it becomes possible that the consultation will be concluded [40]. After reporting an issue with their bowel function, the patient asks a question about normality that indicates their need for further support (lines 35–36). In this instance, and other similar instances, the gyne-oncologist subsequently provides general dietary recommendations (lines 80–81). Their advice to increase fiber intake is introduced as “other dietary things” the patient could do to manage this late effect of cancer treatment, in addition to laxative use (lines 75–83). This instance is an example of how diet-related issues, identified by a patient in response to a gyne-oncologist asking about additional concerns, can lead to a care-related outcome when the patient indicates needing further support with their diet-related issue.

The next fragment is an instance where weight-related talk is sustained and leads to referral to support. This fragment follows the same trajectory as the previous fragment but in the context of a weight-related issue identified by the patient as a stand-alone topic (i.e., not in response to a question from the gyne-oncologist).

Fragment 2 [G03, P26, 03:18–03:51]

**Table:**
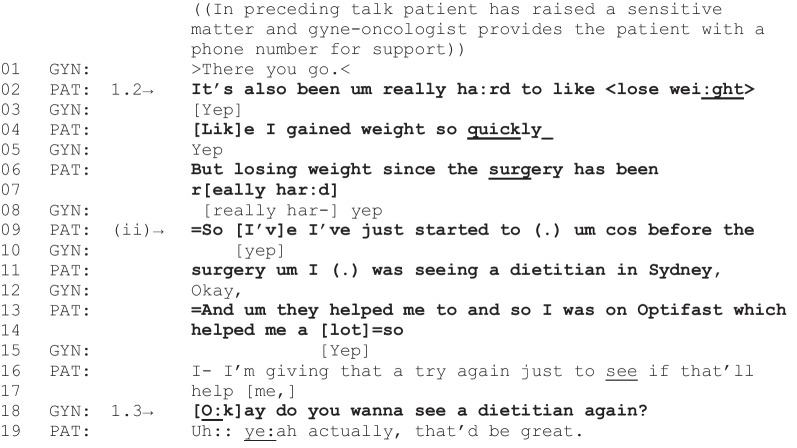


*Patient characteristics: endometrial cancer, obese, intending to lose weight*

In initiating a discussion about weight (line 2), the patient accomplishes two key actions that appear to facilitate care-related outcomes: the patient identifies an issue (weight gain post-treatment) (line 4), and a need for further support (losing weight has been “really hard,” a sentiment repeated twice in lines 2 and 6–7). The gyne-oncologist’s overlap of “really hard” in line 8 is a collaborative completion anticipating the need for further support [41]. The patient then reports their previous experience of seeing a dietitian and indicates a positive outcome of this encounter (lines 9–17). The patient’s stance towards referral to a dietitian as a potential solution to their difficulty losing weight creates an opportunity for the gyne-oncologist to utilize this solution: “Okay do you wanna see a dietitian again?” (line 18), an offer that is accepted by the patient (line 19). When referrals were made in the consultations recorded for this study, these were not always to dietitian services; in another similar instance, a patient with obesity suggested they would benefit from seeing a psychologist to help them with their “triggers” for emotional eating and a referral was subsequently offered by the gyne-oncologist and accepted by the patient. Returning to this instance, although the diet-related issue was raised by the patient as a stand-alone topic and not in response to a query from the gyne-oncologist, it follows the same trajectory as Fragment 1 with the delivery of a care-related outcome when the patient indicates a need for further support.

The next fragment involves a discussion about weight loss that leads to behavior change counseling.

Fragment 3 [G01, P04, 03:48–06:05]

**Table:**
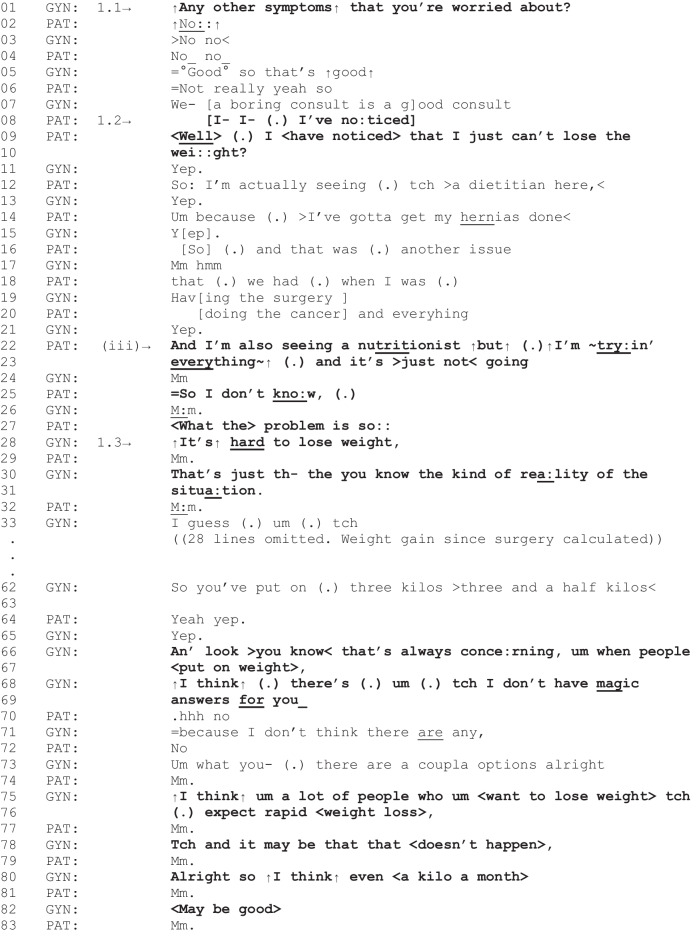


*Patient characteristics: endometrial cancer, obese, intending to lose weight*

In Fragment 3, the patient brings up difficulty losing weight in response to the gyne-oncologist’s inquiry about additional concerns (line 1). Like Fragment 1, additional concerns were not initially elicited (lines 2–7) but are raised shortly thereafter: “Well I have noticed that I just can’t lose the weight” (lines 8–10). In this instance, the patient continues to explain how their problem persists despite different weight loss attempts, including use of professional support (lines 12–23). These comments diminish the relevance of general dietary recommendations or referral to support. However, the patient’s summary, “So I don’t know what the problem is so” (lines 25–27), finishes with a “trail off” conjunction (“so”), indicating their talk is possibly complete and transition of talk to the gyne-oncologist would be a relevant next turn [42]. The gyne-oncologist responds by acknowledging the patient’s experience (lines 28–31) and later adjusting expectations for weight loss (lines 66–82). Through this talk, the gyne-oncologist provides behavior change counseling that focuses on the difficulty of losing weight and the value of gradual weight loss. The delicacy involved in discussing the patient’s weight is interactionally demonstrated through the gyne-oncologist’s frequent use of qualifiers and hedges (e.g., “you know,” “I think,” “I guess”) [43]. Furthermore, the gyne-oncologist uses indirect language by referring to “people” in general, rather than the patient themselves (lines 67, 75). However, as reported in primary care settings, this non-personal approach, designed to avoid straining the doctor-patient relationship, can produce minimal acknowledgement from patients [44]. This is observed in this fragment with the patient’s minimal responses to the gyne-oncologist’s weight management counseling (e.g., “mm” at lines 29, 32, 74, 77, 79, 81, and 83). When spoken with falling intonation, as in this fragment, “mm” can indicate weak acknowledgement of prior talk and that the speaker of these utterances has nothing further to add [45]. In this case, the gyne-oncologist is affirming what the patient has already made clear, for example, “I just can’t lose the weight” in lines 9–10 is affirmed by the gyne-oncologist in line 28 (“It’s hard to lose weight”). Although this fragment suggests that this approach to behavior change counseling may not be effective in motivating patients towards behavior change, this study was not designed to assess post-consultation outcomes of diet, nutrition, or weight-related talk during outpatient follow-up. Nevertheless, these conversations are recommended as part of optimal survivorship care [5], and this instance demonstrates the delivery of a care-related outcome when the patient raises an issue that is relevant to the current clinical activity followed by a need for further support.

### Diet, nutrition, or weight-related talk sustained but no care-related outcomes accomplished

Fragments 4 and 5 illustrate instances where diet, nutrition, or weight-related talk continued beyond initiation but did not culminate in care-related outcomes. The following sequence of actions was observed in these and other instances that followed the same trajectory (beginning at 2.2 if patient initiated):

2.1 Gyne-oncologist inquires about potential treatment late effects, signs of cancer recurrence, or additional concerns.
2.2 Patient reports a diet, nutrition, or weight-related issue post-treatment that is directly relevant to the clinical activity initiated by the gyne-oncologist’s inquiry or introduced as a stand-alone topic. The patient then continues to report:
i. Being resigned to the status quo, and/or
ii. Self-managing the issue.
2.3 Patient or gyne-oncologist transition talk to a different subject.

The next fragment is an instance where diet-related talk is sustained, but the patient indicates being resigned to their status quo. Following this, no care-related outcome is accomplished. The fragment begins with the gyne-oncologist inquiring about the patient’s bowel and bladder function as part of the clinical activity of monitoring for late effects of cancer treatment.

Fragment 4 [G03, P19, 00:21–00:46]

**Table:**
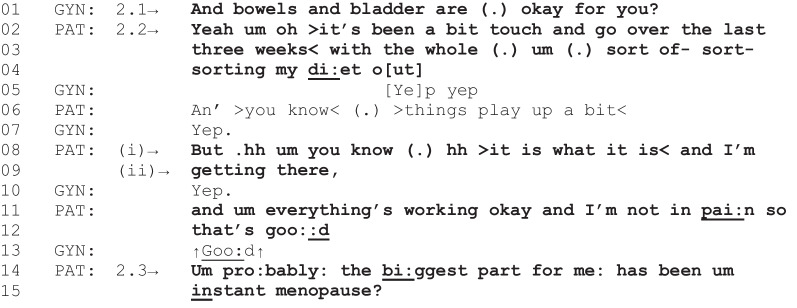


*Patient characteristics: endometrial cancer, obese, intending to lose weight*

In response to the gyne-oncologist’s inquiry about bowel and bladder function, the patient in Fragment 4 identifies that diet is relevant to their bowel issue (lines 1–4). However, unlike Fragments 1 to 3, where patients’ problem identifications are followed by expressing need for further support, in this instance, the patient indicates they are resigned to their current state of function and are self-managing the issue. For example, in line 8, the patient’s resignation is demonstrated through their audible inhalation and exhalation (i.e., a sigh) followed by “it is what it is” [46]. The patient’s claim to be self-managing their bowel and bladder function is accomplished through several practices. First, the patient does not share any specific information about the nature of their problem that has been “a bit touch and go” (line 2). Instead, the patient uses “you know” in lines 6 and 8, proposing shared knowledge of what the issue may be and projecting agreement from the gyne-oncologist [47]. Second, through their statement, “I’m getting there and everything’s working okay” (lines 8–11), the patient accepts the candidate answer produced by the gyne-oncologist in their initial query in line 1, “and bowels and bladder are okay for you?” [48]. This query from the gyne-oncologist demonstrates optimization, a fundamental principle of medical questioning that favors the confirmation of positive health outcomes from patients, allowing the information-gathering part of the consultation to proceed in a timely manner [49]. Thus, although bowel function is reportable as an issue, the patient depicts it as not beyond what is expected and manageable. Third, the patient finishes their turn with a positive assessment of their situation, “and I’m not in pain so that’s good” (lines 11–12). This statement serves as an optimistic projection, a feature used in conversation about problems to move attention away from the issue presently being discussed and onto a new topic [50]. In this instance, the patient’s optimistic projection is accepted by the gyne-oncologist (“Good” in line 13), and the patient transitions talk to their menopausal symptoms indicating the relative priority of this matter over prior talk (lines 14–15). Thus, although the patient identified a diet-related issue that was relevant to the current clinical activity, their indication of being resigned to the status quo and self-managing the issue minimized the relevance of a care-related outcome in this instance.

The next fragment is another instance where diet and weight-related talk is sustained but does not lead to a care-related outcome. In this fragment, a care-related outcome is not made relevant because the patient reports ongoing self-management of their weight.

Fragment 5 [G03, P16, 00:36–02:32]

**Table:**
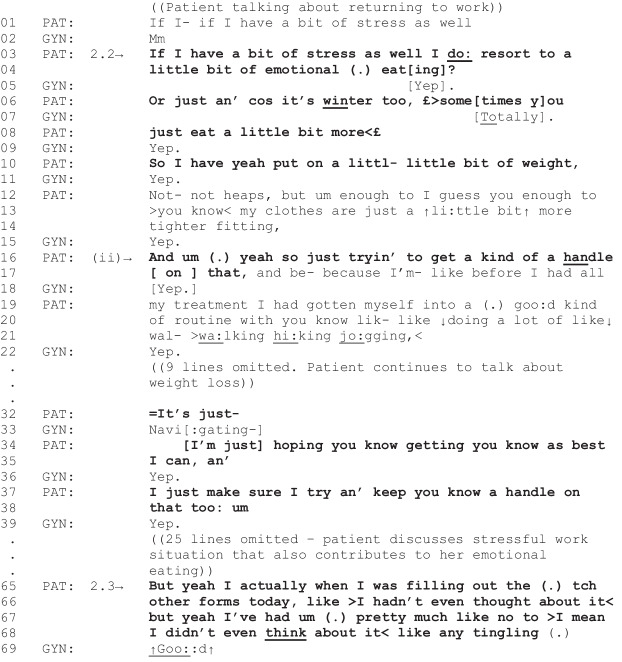


*Patient characteristics: ovarian cancer, overweight, intending to lose weight*

In Fragment 5, the patient identifies an issue with weight gain post-treatment as a stand-alone topic (lines 1–10). The patient follows this problem identification by reporting they are self-managing the issue: “yeah so just tryin’ to get a kind of handle on that” (lines 16–17, later repeated in lines 37–38). The patient’s self-management is further supported by their account of the underlying causes of their weight gain post-treatment. For example, they attribute weight gain to “emotional eating” (line 4), and eating more during winter (lines 6–8), while also explaining their capacity to self-manage their weight: “…before I had all my treatment, I had gotten myself into a good kind of routine… doing a lot of walking, hiking, jogging, and I’d lost a bit of weight before then too” (lines 17–21). Similar to the previous fragment, the patient’s use of “you know” (lines 34 and 37) projects an aligning response to their narrative from the gyne-oncologist (see lines 36 and 39) [47]. Additionally, the patient’s optimistic projection in line 34 (“I’m just hoping…”) implicates closing down of this spate of talk about their weight issue [50]. The instance finishes with the patient transitioning talk to their neuropathy symptoms, signaling this distinct change in topic with “actually” (line 65) [51]. Similar to the previous fragment, this fragment demonstrates another instance where the diet, nutrition, or weight-related topic raised by the patient was relevant to the current clinical activity, but the patient indicated they were self-managing the issue so, appropriately in these instances, no care-related outcome was observed.

### Diet, nutrition, or weight-related talk not substantially sustained beyond initiation

Fragments 6 and 7 illustrate instances where diet, nutrition, or weight-related talk were not substantially sustained beyond initiation. The following sequence of actions was observed in these and other instances that followed the same trajectory (beginning at 3.2 if patient initiated):

3.1 Gyne-oncologist inquires about changes in weight as part of monitoring treatment late effects or signs of cancer recurrence.
3.2  Patient reports trying, wanting, or needing to lose weight, or reports a concern about weight gain post-treatment. However, intentional weight loss is not directly relevant to the clinical activity initiated by the gyne-oncologist’s inquiry.
3.3  Gyne-oncologist does not orient to weight loss as an immediate priority and returns to their broader clinical activity of monitoring for the late effects of treatment or signs of cancer recurrence, or initiates discussion about future clinical surveillance.

The following fragment is an instance where weight-related talk is not substantially sustained following its introduction by the patient. The fragment begins with the gyne-oncologist changing the patient’s hormone replacement therapy, following an assessment of potential risks with the current medication due to the patient’s cancer type and obesity.

Fragment 6 [G01, P08, 31:38–32:06]

**Table:**
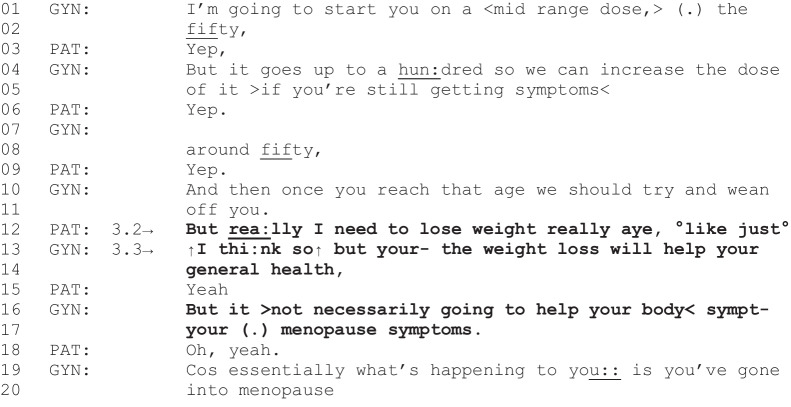


*Patient characteristics: endometrial cancer, obese, intending to lose weight*

After the gyne-oncologist explains the recommended dose for the new hormone replacement medication (lines 1–11), the patient responds, “But really I need to lose weight, really aye” (line 12). In doing so, the patient introduces weight loss as a potentially relevant discussion and seeks agreement from the gyne-oncologist (“really aye”). Although the gyne-oncologist initially provides a weak agreement to the patient’s assessment of their need to lose weight (“I think so”), the gyne-oncologist does not orient to weight loss as being immediately relevant to the current discussion (lines 13–14). The gyne-oncologist accounts for this by identifying that weight loss would help the patient’s “general health” but would not necessarily help their menopause symptoms (lines 16–17), the purpose of the current clinical activity. The gyne-oncologist then supersedes the potential weight discussion with talk about menopause, refocusing the conversation to the primary clinical activity (lines 19–20). Thus, unlike the previous two trajectories where diet, nutrition, or weight-related talk continued beyond initiation, talk in this instance was not pursued further as the topic raised by the patient was not ostensibly relevant to the clinical activity in progress. Nevertheless, this instance demonstrates a potential missed opportunity for weight-related support since the patient displayed readiness for this discussion.

The next fragment begins with the gyne-oncologist directly asking a patient about their weight and exercise (lines 1–3). Thus, unlike Fragment 6, where discussion about intentional weight loss was not considered to be directly relevant to the clinical activity being undertaken at the time, in this instance, discussion about intentional weight loss could be directly relevant in response to the gyne-oncologist’s inquiry. However, the gyne-oncologist then clarifies that their weight question is in relation to concerns about weight loss (lines 8–9).

Fragment 7 [G03, P17, C03, 03:29–04:12]

**Table:**
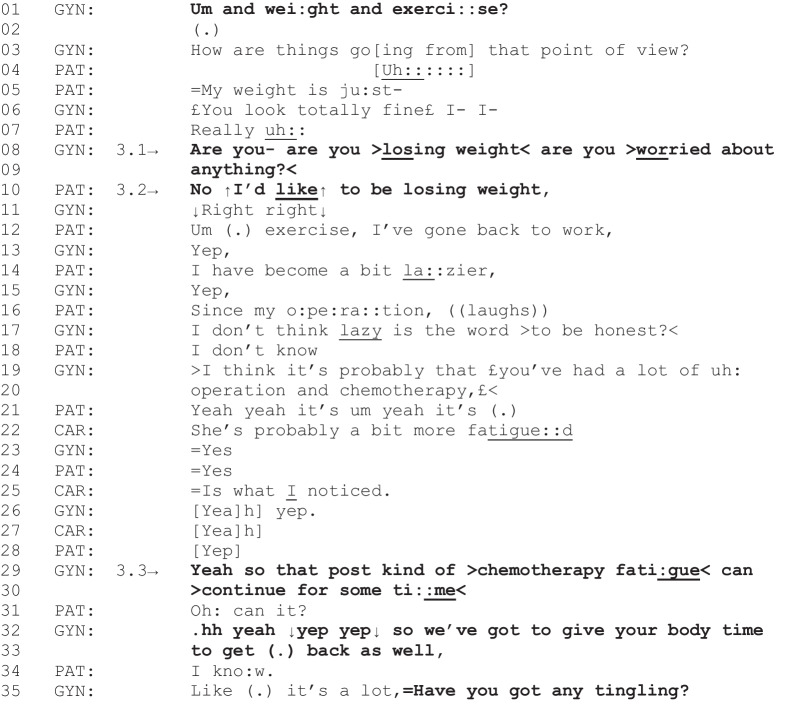


*Patient characteristics: ovarian cancer, overweight, intending to lose weight*

The patient initially responds to the gyne-oncologist’s inquiry with a groan (lines 4 and 7), indicating potential difficulty with their weight and exercise. The patient then reports they would like to be losing weight (line 10). However, it becomes apparent in the immediately ensuing talk that the patient is still experiencing significant fatigue post-treatment, and this is further confirmed by their caregiver (lines 14–28). Thus, weight loss attempts now may not be effective. Consequently, despite their original inquiry, the gyne-oncologist does not orient to intentional weight loss as an immediate priority for the patient. The gyne-oncologist accounts for this by indicating that chemotherapy fatigue can continue “for some time” (lines 29–30) and “we’ve got to give your body time to get back as well” (lines 32–33). The patient’s responses in this segment culminate in a display of acceptance of what the gyne-oncologist is saying with “I know” (line 34) [52], and the gyne-oncologist returns to the clinical activity of monitoring for late effects of treatment (line 35), superseding further weight loss talk. Thus, although weight-related talk was initially relevant to the current clinical activity, when it becomes apparent that the patient is not losing weight unintentionally but is still experiencing late effects from the cancer treatment, talk about intentional weight loss is not pursued further by the gyne-oncologist. However, like the previous fragment, this is a potential missed opportunity for weight-related support since the patient had indicated they would like to be losing weight and the talk concluded with the patient accepting, but not necessarily agreeing with, the gyne-oncologist’s stance that weight loss is not an immediate priority.

## Discussion and conclusion

### Discussion

This conversation analytic study examined how diet, nutrition, and body weight-related topics are discussed during outpatient follow-up after treatment for gynecological cancer. In sequences of talk about diet, nutrition, or weight, only one sequential trajectory resulted in general dietary recommendations, referral to support, or behavior change counseling. Patients who received one of these care-related outcomes explicitly communicated with the gyne-oncologist about their need for further support with their diet, nutrition, or weight-related issue. This aligns with previous research in primary care, where patients were more likely to receive diet or weight-related information and referral to support if they identified their current status as problematic [27] or explicitly communicated their readiness to change [26]. Additionally, cancer care clinicians report that their promotion of healthy dietary changes to cancer survivors is influenced by their perceptions of the patient’s motivation and barriers to change [53]. Thus, our study findings, along with previous research, suggest that patients seeking diet, nutrition, or weight-related information or referral from their gyne-oncologist post-treatment may only receive such support if they explicitly request it. This highlights a need for better integrating diet, nutrition, and weight-related talk into routine survivorship care so that these needs can be identified and addressed independent of patient assertion of their need for support.

We also identified that the continuation of diet, nutrition, and weight-related talk, which occasioned the possibility of care-related outcomes, was dependent on the apparent relevance of the topic to the clinical activity being undertaken at the time. When patients raised an issue that was not directly relevant to the current clinical activity, diet, nutrition, or weight-related talk was not pursued further in conversation. A possible explanation for this is the concept of “activity contamination,” as described by Whalen and colleagues [54]. In their analysis of phone calls to emergency services, they identified that call receivers had several priority tasks they needed to complete in a timely manner in order to mobilize prompt medical care [54]. When the caller raised a topic that was not directly relevant to completing these priority tasks, the topic was not pursued further by the call receiver; this avoided a potential change in the trajectory of the conversation that could jeopardize the mobilization of prompt medical care [54].

Although our analysis was conducted in a different setting to the emergency services calls studied by Whalen and colleagues [54], gyne-oncologists must also complete multiple priority tasks within a time-limited consultation. These priority tasks include assessing and managing treatment sequelae or late effects, checking for signs and symptoms of recurrent disease and providing information for self-monitoring, establishing a pathway for future clinical surveillance, and discussing healthy lifestyle behaviors, such as diet, physical activity, smoking cessation, and weight management [4, 5]. Thus, completing these clinical activities in a timely manner necessitates avoiding “activity contamination” that may substantially alter the trajectory of the consultation. Indeed, in a national survey of gyne-oncology clinicians, the most important barrier and facilitator to providing supportive care was sufficient time to discuss these issues with patients [15].

The avoidance of activity contamination in our study was interactionally demonstrated when patients raised the topic of intentional weight loss when it was not apparently relevant to the clinical activity being undertaken at the time; for example, when the gyne-oncologist was initiating a change to hormone replacement medication or when the patient was still experiencing late effects of their cancer treatment. In these instances, the gyne-oncologist did not orient to intentional weight loss as an immediate priority and superseded further weight discussion by returning to their original clinical activity or initiating a new activity. This avoided a potential change in the trajectory of the conversation that could hinder the progression of important clinical tasks. However, it is notable that, in instances where weight talk did not continue further, patients had still identified an issue or concern with their weight gain post-treatment. Therefore, it is possible that some patients left the consultation with unmet information and support needs for weight management. A recent scoping review identified that unmet needs for diet-related information in healthcare settings often led to cancer survivors seeking that information elsewhere, commonly from online and interpersonal sources [20]. This highlights the importance of meeting patients’ needs for survivorship care information to ensure cancer survivors have access to appropriate and evidence-based support.

Overall, these findings suggest that additional avenues for dietary needs assessment and referral to support may be needed to ensure consistent delivery of dietary care to women who have completed treatment for gynecological cancer. Future work could develop, implement, and evaluate these avenues, such as integrating screening for diet, nutrition, and weight-related needs with pre-consultation screening for other supportive care needs. Future research could also assess patient outcomes following diet, nutrition, and weight-related communication during post-treatment follow-up.

### Strengths and limitations

This is the first study to provide direct observational evidence for how diet, nutrition, and weight-related discussions unfold during outpatient follow-up after treatment for gynecological cancer, and the challenges associated with enacting survivorship care guidelines for these discussions in clinical practice. An often-cited limitation of observational studies of people’s behavior is the “Hawthorne Effect,” whereby people alter their behavior when being observed [55]. However, previous research indicates that this effect is not a significant limitation for communication research, because participants’ awareness that they are being observed has minimal impact on their communication behavior [56]. Additionally, participants were unaware of the diet, nutrition, and weight-related focus of this study when the consultations were being recorded.

This study also has some limitations. It is not known whether those who declined to participate differed to study participants in relation to sociodemographic and health characteristics as this information was not collected. The cross-sectional design of this study means diet, nutrition, or weight-related conversations in previous or subsequent outpatient visits were not captured. Thus, the talk observed in this analysis may not fully represent participants’ diet, nutrition, and weight-related needs or the participation of accompanying family or friends in these conversations. For example, none of the consultations recorded discussed ongoing symptoms affecting food intake (e.g., poor appetite, early satiety), despite one-third of the sample reporting multiple nutrition impact symptoms post-treatment (see Online Resource 2). Furthermore, it is not known what discussions occurred in other settings (e.g., with general practitioners or allied health). The use of audio recordings means that multimodal dimensions of interaction (e.g., body language, eye gaze, facial expressions) were not able to be included in this analysis [29]. Finally, the study was conducted in a publicly funded hospital; it is not known whether the same communication practices would be observed in privately funded care settings.

### Conclusion

This conversation analytic study of outpatient consultations after treatment for gynecological cancer identified that diet, nutrition, or weight-related talk continued beyond initiation if it was ostensibly relevant to the clinical activity being undertaken at the time. Patients who then explicitly indicated a need for further support with their diet, nutrition, or weight-related issue received general dietary recommendations, referral to support, or behavior change counseling. Diet, nutrition, or weight-related topics raised by patients that did not align with the clinical activity being undertaken at the time were not pursued further in conversation. Although the possibility of activity contamination may account for this, these instances are nonetheless missed opportunities for the provision of dietary information and support after treatment for gynecological cancer. Additional avenues for dietary needs assessment and referral to support may be needed, as this may not be optimally achieved during outpatient consultations after treatment for gynecological cancer.

### Electronic supplementary material

Below is the link to the electronic supplementary material.

Supplementary file1 (DOCX 43.1 KB)

## Data Availability

Transcripts of the datasets generated and analyzed during the current study are available from the corresponding author upon reasonable request.

## Appendix

**Table 1:** Conversation analytic transcriptions

Temporal dimensions	
Wo**[**rd**]** W**[**or**]**d	Overlapping speech
Word**=**word	Latching, or absence of discernible silence between two utterances
Word (4.0) word	Silence, measured to the nearest second
Vocal conduct	
Word (.) word	A pause less than one second in length
Word.	Falling intonation at the end of a unit of talk
Word**,**	Slightly rising intonation
Word**?**	Rising intonation
Word**_**	Level intonation
**Wo**rd	Emphasis
Wo**:::**rd	Stretching of the immediately preceding sound, with multiple colons representing prolonged stretching
W**o:****:**rd	Shift in pitch, with rising pitch on the underlined component followed by falling pitch on the colon component that is not underlined
Wo**:****:**rd	Shift in pitch, with rising intonation on the underlined colon component
**↑**Word**↑**	Sharp increased pitch shift
**↓**Word**↓**	Sharp decreased pitch shift
**°**Word**°**	Talk produced at a lower volume than surrounding utterances by the same speaker
** > **Word** < **	Talk produced at a faster pace than surrounding talk
** < **Word** > **	Talk produced at a slower pace than surrounding talk
Wor**-**	Abrupt termination in the pronunciation of the preceding sound
**£**Word**£**	Smile voice
** ~ **Word** ~ **	Tremulous voice
hhh	Audible exhalation, with more letters indicating longer exhalation
.hhh	Audible inhalation, with more letters indicating longer inhalation
.tch	Audible tongue click
**((**Description**))**	Used to provide explanation of omitted lines of talk
