# Supplementary material for: Discussing diet, nutrition, and body weight after treatment for gynecological cancer: a conversation analytic study of outpatient consultations
Source: J Cancer Surviv. 2023 Mar 10;18(3):1016–31. doi: 10.1007/s11764-023-01345-w (PMC11081991; doi:10.1007/s11764-023-01345-w)
Supplement: Supplementary file 1 — Supplementary file1 (DOCX 43.1 KB) [file 11764_2023_1345_MOESM1_ESM.docx]

**Online Resource 1:** Participant flow chart for patients included in the conversation analytic study of diet, nutrition, or weight-related discussions during outpatient follow-up after treatment for gynecological cancer

Seen by a clinician not participating in the study, N=3

Recording failed, N=1

Declined to participate

N=12

Did not attend outpatient appointment

N=11

Consultations recorded

N=30

Consented to participate

N=34

Attended outpatient appointment

N=46

Eligible patients

N=57

**Online Resource 2:** Characteristics of patients included in the conversation analytic study of diet, nutrition, or weight-related discussions during outpatient follow-up after treatment for gynecological cancer by cancer type

|  | **Total sample^a^ (n=30)** | **Ovarian**  **(n=11)** | **Endometrial**  **(n=19)** |
| --- | --- | --- | --- |
| **Clinical factors** |  |  |  |
| Stage of disease at diagnosis |  |  |  |
| Stage I-II | 16 (53%) | 4 (36%) | 12 (63%) |
| Stage III-IV | 14 (47%) | 7 (64%) | 7 (37%) |
| Treatment received^b^ |  |  |  |
| Surgery | 30 (100%) | 11 (100%) | 19 (100%) |
| Chemotherapy | 17 (57%) | 10 (91%) | 7 (37%) |
| Radiation therapy | 11 (37%) | 0 (0%) | 11 (58%) |
| Months since treatment completion |  |  |  |
| ≤6 months | 17 (57%) | 7 (64%) | 10 (53%) |
| >6 months | 13 (43%) | 4 (36%) | 9 (47%) |
| Treatment response |  |  |  |
| Complete | 28 (93%) | 9 (82%) | 19 (100%) |
| Incomplete (stable/progressive) | 2 (7%) | 2 (18%) | 0 (0%) |
| **Socio-demographic factors** |  |  |  |
| Age (years) |  |  |  |
| <65 | 21 (70%) | 6 (55%) | 15 (79%) |
| ≥65 | 9 (30%) | 5 (45%) | 4 (21%) |
| Education |  |  |  |
| High school or less | 16 (55%) | 6 (60%) | 10 (53%) |
| Vocational / University | 13 (45%) | 4 (40%) | 9 (47%) |
| Body Mass Index category |  |  |  |
| Normal | 3 (10%) | 2 (18%) | 1 (5%) |
| Overweight | 7 (23%) | 5 (45%) | 2 (11%) |
| Obese | 20 (67%) | 4 (36%) | 16 (84%) |
| Current weight intentions |  |  |  |
| Trying to gain weight | 0 (0%) | 0 (0%) | 0 (0%) |
| Trying to lose weight | 19 (66%) | 6 (60%) | 13 (68%) |
| Neither | 10 (34%) | 4 (40%) | 6 (32%) |
| **Health behaviors and wellbeing** |  |  |  |
| Number of symptoms affecting food intake in the past 2 weeks | | | |
| <3 | 19 (63%) | 6 (55%) | 13 (68%) |
| ≥3 | 11 (37%) | 5 (45%) | 6 (32%) |
| Food intake during the past month |  |  |  |
| More than usual | 9 (31%) | 2 (20%) | 7 (37%) |
| Less than usual | 7 (24%) | 2 (20%) | 5 (26%) |
| Unchanged | 13 (45%) | 6 (60%) | 7 (37%) |
| Usual fruit intake (serves/day) |  |  |  |
| <2 | 15 (52%) | 6 (60%) | 9 (47%) |
| ≥2 | 14 (48%) | 4 (40%) | 10 (53%) |
| Usual vegetable intake (serves/day) | |  |  |
| <5 | 27 (93%) | 9 (90%) | 18 (95%) |
| ≥5 | 2 (7%) | 1 (10%) | 1 (5%) |
| Current activity and function |  |  |  |
| Normal with no limitations | 15 (52%) | 3 (30%) | 12 (63%) |
| Not normal self, but still mobile | 12 (41%) | 6 (60%) | 6 (32%) |
| Not feeling up to most things | 1 (3%) | 1 (10%) | 0 (0%) |
| Able to do little activity | 1 (3%) | 0 (0%) | 1 (5%) |
| Bed-ridden | 0 (0%) | 0 (0%) | 0 (0%) |
| Smoking status |  |  |  |
| Current | 2 (7%) | 0 (0%) | 2 (11%) |
| Former | 17 (59%) | 6 (60%) | 11 (58%) |
| Never | 10 (34%) | 4 (40%) | 6 (32%) |
| **Dietary follow-up** |  |  |  |
| Diet or weight-related discussion at follow-up | |  |  |
| Yes | 18 (60%) | 6 (55%) | 12 (63%) |
| No | 12 (40%) | 5 (45%) | 7 (37%) |
| Dietary care post-treatment |  |  |  |
| Dietitian or other nutrition professional | 5 (17%) | 1 (10%) | 4 (21%) |
| None | 24 (83%) | 9 (90%) | 15 (79%) |
| Seeking dietary information or support at follow-up | | |  |
| Yes | 10 (34%) | 0 (0%) | 10 (53%) |
| No | 19 (66%) | 10 (100%) | 9 (47%) |
| Talk to gyne-oncologist about diet or weight-related needs or concerns | | | |
| Yes | 18 (62%) | 5 (50%) | 13 (68%) |
| No | 11 (38%) | 5 (50%) | 6 (32%) |
| Preferences for diet and weight discussions^b^ | |  |  |
| No preference | 14 (48%) | 8 (80%) | 6 (32%) |
| General Practitioner | 11 (37%) | 1 (10%) | 10 (53%) |
| Dietitian | 6 (20%) | 1 (10%) | 5 (26%) |

^a^ Total sample N=29 where data missing for 1 ovarian cancer patient.
^b^ Multiple responses accepted, adds to >100%. Participants who reported they would talk to their gyne-oncologist about diet or weight-related needs or concerns also answered the question about preferences.

**Online Resource 3:** Symptoms affecting food intake reported by patients in the conversation analytic study of diet, nutrition, or weight-related discussions during outpatient follow-up after treatment for gynecological cancer by cancer type

|  | **Ovarian**  **(n=10)^a^** | **Endometrial**  **(n=19)** | **Total sample**  **(n=29)^a^** |
| --- | --- | --- | --- |
| No problems eating | 0 (0%) | 5 (26%) | 5 (17%) |
| Poor appetite | 1 (10%) | 5 (26%) | 6 (21%) |
| Nausea | 1 (10%) | 4 (21%) | 5 (17%) |
| Vomiting | 0 (0%) | 0 (0%) | 0 (0%) |
| Constipation | 5 (50%) | 1 (5%) | 6 (21%) |
| Diarrhea | 1 (10%) | 2 (11%) | 3 (10%) |
| Dry mouth | 4 (40%) | 3 (16%) | 7 (24%) |
| Mouth sores | 0 (0%) | 0 (0%) | 0 (0%) |
| Sensitivity to smells | 1 (10%) | 2 (11%) | 3 (10%) |
| Altered taste | 1 (10%) | 1 (5%) | 2 (7%) |
| Fatigue | 8 (80%) | 11 (58%) | 19 (66%) |
| Early satiety | 6 (60%) | 6 (32%) | 12 (41%) |
| Problems swallowing | 1 (10%) | 1 (5%) | 2 (7%) |
| Pain | 0 (0%) | 0 (0%) | 0 (0%) |
| Other (e.g., stress, tingling / numbness) | 1 (10%) | 1 (5%) | 2 (7%) |

**^a^** 1 ovarian cancer patient missing data for symptoms affecting food intake. Percentages calculated
excluding missing data.
